# Towards robust and versatile single nanoparticle fiducial markers for correlative light and electron microscopy

**DOI:** 10.1111/jmi.12778

**Published:** 2019-01-16

**Authors:** J.J.H.A. VAN HEST, A.V. AGRONSKAIA, J. FOKKEMA, F. MONTANARELLA, A. GREGORIO PUIG, C. DE MELLO DONEGA, A. MEIJERINK, G.A. BLAB, H.C. GERRITSEN

**Affiliations:** ^1^ Condensed Matter and Interfaces, Debye Institute for Nanomaterials Science Utrecht University Utrecht The Netherlands; ^2^ Molecular Biophysics, Debye Institute for Nanomaterials Science Utrecht University Utrecht The Netherlands; ^3^ Soft Condensed Matter, Debye Institute for Nanomaterials Science Utrecht University Utrecht The Netherlands

**Keywords:** Correlative microscopy, fiducial markers, integrated correlative microscopy, lanthanides, luminescence

## Abstract

Fiducial markers are used in correlated light and electron microscopy (CLEM) to enable accurate overlaying of fluorescence and electron microscopy images. Currently used fiducial markers, e.g. dye‐labelled nanoparticles and quantum dots, suffer from irreversible quenching of the luminescence after electron beam exposure. This limits their use in CLEM, since samples have to be studied with light microscopy before the sample can be studied with electron microscopy. Robust fiducial markers, i.e. luminescent labels that can (partially) withstand electron bombardment, are interesting because of the recent development of integrated CLEM microscopes. In addition, nonintegrated CLEM setups may benefit from such fiducial markers. Such markers would allow switching back from EM to LM and are not available yet.

Here, we investigate the robustness of various luminescent nanoparticles (NPs) that have good contrast in electron microscopy; 130 nm gold‐core rhodamine B‐labelled silica particles, 15 nm CdSe/CdS/ZnS core–shell–shell quantum dots (QDs) and 230 nm Y_2_O_3_:Eu^3+^ particles. Robustness is studied by measuring the luminescence of (single) NPs after various cycles of electron beam exposure. The gold‐core rhodamine B‐labelled silica NPs and QDs are quenched after a single exposure to 60 ke^−^ nm^–2^ with an energy of 120 keV, while Y_2_O_3_:Eu^3+^ NPs are robust and still show luminescence after five doses of 60 ke^−^ nm^–2^. In addition, the luminescence intensity of Y_2_O_3_:Eu^3+^ NPs is investigated as function of electron dose for various electron fluxes. The luminescence intensity initially drops to a constant value well above the single particle detection limit. The intensity loss does not depend on the electron flux, but on the total electron dose. The results indicate that Y_2_O_3_:Eu^3+^ NPs are promising as robust fiducial marker in CLEM.

**Lay Description:**

Luminescent particles are used as fiducial markers in correlative light and electron microscopy (CLEM) to enable accurate overlaying of fluorescence and electron microscopy images. The currently used fiducial markers, e.g. dyes and quantum dots, loose their luminescence after exposure to the electron beam of the electron microscope. This limits their use in CLEM, since samples have to be studied with light microscopy before the sample can be studied with electron microscopy. Robust fiducial markers, i.e. luminescent labels that can withstand electron exposure, are interesting because of recent developments in integrated CLEM microscopes. Also nonintegrated CLEM setups may benefit from such fiducial markers. Such markers would allow for switching back to fluorescence imaging after the recording of electron microscopy imaging and are not available yet.

Here, we investigate the robustness of various luminescent nanoparticles (NPs) that have good contrast in electron microscopy; dye‐labelled silica particles, quantum dots and lanthanide‐doped inorganic particles. Robustness is studied by measuring the luminescence of (single) NPs after various cycles of electron beam exposure. The dye‐labelled silica NPs and QDs are quenched after a single exposure to 60 ke^−^ nm^–2^ with an energy of 120 keV, while lanthanide‐doped inorganic NPs are robust and still show luminescence after five doses of 60 ke^−^ nm^–2^. In addition, the luminescence intensity of lanthanide‐doped inorganic NPs is investigated as function of electron dose for various electron fluxes. The luminescence intensity initially drops to a constant value well above the single particle detection limit. The intensity loss does not depend on the electron flux, but on the total electron dose. The results indicate that lanthanide‐doped NPs are promising as robust fiducial marker in CLEM.

## Introduction

Correlative light and electron microscopy (CLEM) is a powerful tool that combines the high sensitivity and large field of view of fluorescence (light) microscopy with the high resolution of electron microscopy to study structures and molecular distributions with nanometre resolution. The use of integrated CLEM is relatively new and is mainly used to investigate biological samples (Su *et al*., 2010; De Boer *et al*., 2015), but also finds application in material science (Karreman *et al*., 2012; Hendriks *et al*., 2018). Fiducial markers which can be detected with both light and electron microscopy can be used to correlate the images obtained with both microscopes. Various types of fiducial markers have been employed over the past decades both in clustered and single particle form (Sosinky *et al*., 2007; Su *et al*., 2010; Schellenberger *et al*., 2014).

Commonly used fiducial markers are organic dyes embedded in or attached to particles which have suitable contrast in electron microscopy, such as latex beads or gold NPs (Schmued & Snavely, 1993; Agronskaia *et al*., 2008; Kukulski *et al*, 2011). Quantum dots (QDs) have also been used as label in light microscopy and as fiducial marker in CLEM (Nisman *et al*., 2004; Giepmans *et al*., 2005; Deerink, 2008). A disadvantage of the organic dyes and QDs as fiducial markers is the limited stability to photobleaching and electron beam exposure. Photobleaching is especially a problem for organic dyes, while QDs are more stable (Dubertret *et al*., 2002; Resch‐Genger *et al*., 2008). However, the luminescence of both organic dyes and QDs is irreversibly quenched after exposure to the electron beam (Rodriguez‐Viejo *et al*., 1997; Niitsuma *et al*., 2005). This limits their use in CLEM, since samples have to be investigated with light microscopy before the samples can be studied in more detail with electron microscopy.

Robust fiducial markers, i.e. luminescent labels that can withstand bombardment with electrons, are interesting, especially in view of recent developments in integrated CLEM systems (Zonnevylle *et al*., 2013; De Boer *et al*., 2015). For example, robust fiducial markers can be employed to monitor distortion of biological samples due to exposure to the electron beam. It has been observed that biological specimens suffer from alterations due to electron beam exposure at ambient temperatures, resulting in e.g. shrinking of the sample (Stenn & Bahr, 1970; Zeitler, 1982). Shrinkage as high as 30% has been observed (Luther *et al*., 1988; Kopek *et al*., 2013). Modifications of samples can be monitored by comparing the light microscopy images of luminescent markers before and after electron microscopy imaging of biological samples. Robust luminescent markers that can withstand electron beam exposure offer great perspective in correlative cathode luminescence (CL) and scanning electron microscopy (SEM) experiments. Correlative CL‐SEM studies have been performed previously on Y_2_O_3_:Ln^3+^ NPs of ∼70 nm (Fukushima *et al*., 2016) and on ∼37 nm LuAG:Ce^3+^ NPs (Glenn *et al*., 2012; Garming *et al*., 2017). However, cathode luminescence measurements on LuAG:Ce^3+^ NPs have revealed a fluctuation in intensity in time, possibly due to bleaching or drifting of the sample during electron exposure (Garming *et al*., 2017). Robust particles that exhibit CL are also of interest in CL‐STEM (Kociak *et al*., 2017) and integrated CLEM systems that allow CL detection (Haring *et al*., 2017). Up to now, the robustness of fiducial markers for combined light and electron microscopy has not been studied in detail.

Lanthanide (Ln^3+^) ions incorporated into an inorganic host are promising as robust fiducial marker. Inorganic NPs have a high stability and the luminescence of lanthanide ions is insensitive to local structural changes as the luminescence involves optical transitions within the 4f^n^ shell. The 4f orbitals are shielded by filled outer 5s and 5p orbitals. As a result, the influence of the environment on the intraconfigurational 4f^n^ transitions is small, which gives Ln^3+^ ions several advantages as luminescent marker. First, the chemical and temperature stability of Ln^3+^ luminescence is high (Weber, 1968; Blasse & Grabmaier, 1994). Next, Ln^3+^ ions show characteristic sharp line emissions at characteristic wavelengths (Blasse & Grabmaier, 1994). Consequently, the Ln^3+^ emission can be detected with a high signal to noise ratio by using narrow band filters. In addition, a variety of luminescent labels can be created by changing and combining the type of lanthanide ions incorporated in the host material. The various labels can be distinguished by measuring the emission spectra of the labels. Finally, Ln^3+^ emission usually has a long (ms) lifetime. As a result, the lanthanide luminescence can be discriminated from autofluorescence by performing time‐gated detection (Lu *et al*., 2014).

In this report, the robustness of lanthanide‐doped inorganic NPs, 230 nm Y_2_O_3_:Eu^3+^, as fiducial markers for CLEM is investigated and compared with commonly used fiducial markers, i.e. dye‐labelled NPs and quantum dots (QDs) by measuring the luminescence intensity of (single) NPs after exposure to various electron doses. The luminescence of 130 nm gold‐core rhodamine B‐labelled silica NPs and 15 nm CdSe/CdS/ZnS core–shell–shell QDs is quenched after exposure to a single electron dose of 60 ke^−^ nm^–2^ with energy of 120 keV. In contrast, the Y_2_O_3_:Eu^3+^ NPs are robust and show luminescence after five electron doses of 60 ke^−^ nm^–2^. To further investigate the stability after electron beam exposure, the luminescence intensity of Y_2_O_3_:Eu^3+^ NPs is investigated as function of electron dose at various electron fluxes. The luminescence intensity initially drops, but rapidly reaches a constant intensity of ∼20% of the initial intensity. The luminescence intensity loss is not dependent on the electron flux, but on the total electron dose. The 200 nm diameter Y_2_O_3_:Eu^3+^ NPs retain their bright luminescence after electron doses as high as 66 Me^−^ nm^–2^ at a level that allows single NP detection. The results indicate that Y_2_O_3_:Eu^3+^ NPs are suitable as robust fiducial marker, down to the single particle level.

## Experimental section

### Synthesis of gold‐core rhodamine B‐labelled silica NPs

All the particles described in this paper were synthesised in our laboratory using established methods. The gold‐core rhodamine B‐labelled silica NPs were synthesised as follows. In the first step, 15 nm gold core nanoparticles (NPs) were synthesised according to the Turkevich method (Turkevich *et al*., 1951; Perrault & Chan, 2009). Next, the gold core NPs were coated with poly(vinylpyrrolidone) wt 10 000 according to the method described by Graf *et al*. (2003). The gold‐core NPs were coated with silica based on the Stöber method (Stöber *et al*., 1968). This procedure was modified according to methods described by Imhof *et al*. (1999) and Verhaegh & van Blaaderen (1994) to incorporate rhodamine B into the silica shell. The as‐synthesised gold‐core rhodamine B‐labelled nanoparticles were dispersed in ethanol (27.5 nM). The final size of the particles was determined by TEM and amounted to 133 ± 6 nm.

### Synthesis of 15 nm CdSe/CdS/ZnS core–shell–shell quantum dots

Red emitting CdSe quantum dots (QDs) with a diameter of 3 nm were prepared using a method described by Montanarella *et al*. (Montanarella *et al*., 2017). First, CdS and ZnS shells were grown around the QDs using a method described by Li *et al*. (2003) and Montanarella *et al*. (2017). Next, 10 monolayers of CdS were grown, followed by two monolayers CdZnS and two monolayers ZnS. The as‐synthesised QDs were dispersed in cyclohexane (100 nM). The size of the CdSe/CdS/ZnS core–shell–shell QDs was determined by TEM and was 15 ± 2 nm.

### Synthesis of Y_2_O_3_:Eu^3+^ (5%) NPs

Y_2_O_3_ NPs doped with 5% europium were synthesised using a method described by Sohn *et al*. (2004). A white powder was obtained after synthesis. The size of the as‐synthesised NPs was determined by TEM and amounted to 228 ± 23 nm.

### Spectroscopy

Absorption spectra were recorded for the QDs. The sample for absorption measurements was prepared by diluting the as‐synthesised QD dispersion hundred times with toluene to a concentration of 1 nM. Absorption spectra were recorded on a double beam Perkin–Elmer Lambda 950 UV/Vis spectrometer.

For all samples luminescence spectra were recorded. Samples were prepared by diluting the gold‐core rhodamine B‐labelled silica NPs stock dispersion hundred times with ethanol to a concentration of 0.3 nM and the QDs 100 times with toluene to a concentration of 1 nM. The Y_2_O_3_:Eu^3+^ sample was prepared by dispersing 10 mg NPs in 5 mL ethanol. After sonification, the suspension was diluted hundred times with ethanol to a concentration of 20 μg mL^–1^ and sonicated again. Photoluminescence spectra of all samples were recorded using an Edinburgh Instruments FLS920 fluorescence spectrometer. Emission and excitation spectra were recorded using a 450 W Xe lamp as excitation source and a Hamamatsu R928 PMT detector.

### Integrated light and electron microscopy

Samples for integrated light and electron microscopy measurements were prepared on TEM grids. The gold‐core rhodamine B‐labelled silica NPs stock dispersion (27.5 nM) was diluted with water. Next, the dispersion was sonicated for 10 min and 25 μL of the dispersion was placed on a Formvar coated copper TEM grid. After evaporation of the solvent, the grid was washed with water. The as‐synthesised QD dispersion was diluted 10 000 times with cyclohexane to a concentration of 0.01 nM and dropcasted on a Formvar coated copper TEM‐grid. The Y_2_O_3_:Eu^3+^ samples were prepared by dispersing 10 mg in 5 mL ethanol. After sonification, the suspension was diluted forty times with ethanol to a concentration of 50 μg mL^–1^ and sonicated again. The diluted NP dispersion was dropcasted on a Formvar coated copper TEM grids.

Optical images of the various samples were acquired with a home‐made wide‐field fluorescence microscope based on the setup described in Agronskaia *et al*. (2008). The microscope will be described in detail in a future publication. Briefly, the fluorescence microscope was mounted on one of the side ports of the TEM octagon and orthogonal to the electron optical axis; for optical imaging the sample stage tilt was set to +90°. The microscope was equipped with an Omicron 120 mW 405 nm diode laser (12 W cm^–2^ on sample) and an Omicron 100 mW 532 nm diode laser (25 W cm^–2^ on sample) as excitation sources. The laser light was guided to the sample through a Chroma ZT532rdc single dichroic and a Nikon CF IC EPI Plan ELWD 0.55 NA, 50x air objective. Various bandpass filters were used to select the emission from the samples: a Chroma ET585/65m filter for the gold‐core rhodamine B‐labelled silica NPs; a ET645/75m filter for CdSe/CdS/ZnS core–shell–shell quantum dots; and a Semrock FF01‐610/5m filter for the Y_2_O_3_:Eu^3+^ NPs. The three‐part number in the filter code represents the wavelength in the middle of the band and the last number indicates roughly the band width. The emission was detected with a PCO sCMOS camera PCO 4.2 edge. For typical measurement shown in the figures, 20 frames with a frame time of 3 s were accumulated. The data was analysed using the ThunderStorm Fiji plugin (Ovesný *et al*., 2014). The plugin was used to determine the intensity and background of single particles.

TEM images were obtained with a FEI Tecnai 12 microscope operating at 120 keV equipped with a tungsten filament. The alpha stage tilt for TEM operation was set to 0°. Images were recorded at room temperature with a TVIPS 2048 × 2048 TEMCam‐F214 CCD camera running iTEM software. Electron fluxes of 2, 100 and 1000 ke^−^ nm^–2^ s with an electron energy of 120 keV were used during electron beam exposure. For a typical experiment, the samples were exposed for 30 s to the electron beam.

## Results and discussion

**Figure 1:**
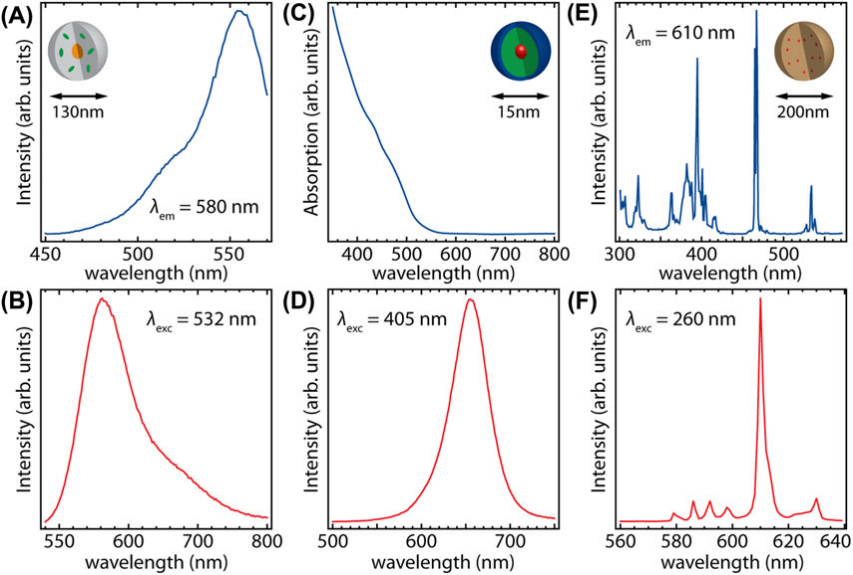
(A) Excitation spectrum (*λ*_em_ = 580 nm) and (B) emission spectrum (*λ*_exc_ = 532 nm) of gold NPs coated with rhodamine B‐labelled silica, (C) absorption and (D) emission spectrum (*λ*_exc_ = 405 nm) of CdSe/CdS/ZnS core–shell–shell QDs, (E) excitation spectrum (*λ*_em_ = 610 nm) and (F) emission spectrum (*λ*_exc_ = 260 nm) of Y_2_O_3_:Eu^3+^ NPs. Insets in (A), (C) and (E) show schematic representations of the NPs.

Three different types of nanoparticles (NPs), i.e. 133 ± 6 nm gold‐core rhodamine B‐labelled silica NPs, 15 ± 2 nm CdSe/CdS/ZnS core–shell–shell quantum dots (QDs) and 228 ± 23 nm Y_2_O_3_:Eu^3+^ NPs, were investigated. The luminescence properties of the various NPs are important, since they determine the measurement settings for the light microscopy measurements. In order to get insight into the luminescence properties of the NPs, absorption, excitation and emission spectra were recorded. In Figure 1, excitation and emission spectra of the gold‐core rhodamine B‐labelled silica and Y_2_O_3_:Eu^3+^ NPs and absorption and emission spectra of the CdSe/CdS/ZnS core–shell–shell QDs are shown. Schematic representations of the various types of NPs are shown in the insets of a, c and e.

The excitation spectrum (*λ*_em_ = 580 nm) of the gold‐core rhodamine B‐labelled silica NPs shows a band centred at 555 nm corresponding to the singlet–singlet (S_0_–S_1_) transition, see Figure 1(A). The emission spectrum (*λ*_exc_ = 532 nm) shows a broad band originating from the reverse transition, see Figure 1(B). The shape and spectral positions of the excitation and emission bands correspond well with the values reported in literature (Gao *et al*., 2009).

The absorption spectrum of CdSe/CdS/ZnS core–shell–shell QDs is shown in Figure 1(C). An onset of absorption is observed around 500 nm, originating from absorption by the CdS shell. The QDs consist of ∼95% CdS, resulting in the dominant absorption feature of CdS in the absorption spectrum. The emission spectrum (*λ*_exc_ = 405 nm) is shown in Figure 1(D). An emission band around 650 nm is observed, originating from the CdSe exciton transition. The spectral positions of absorption and emission bands agree well with the results reported in Dabbousi *et al*. (1997), De Mello Donega (2011) and Montanarella *et al*. (2017).

The excitation spectrum (*λ*_em_ = 610 nm) of Y_2_O_3_:Eu^3+^ NPs is shown in Figure 1(E). Sharp excitation peaks resulting from intraconfigurational transitions to higher energy 4f^6^ levels are observed. The excitation lines are assigned to ^7^F_0,1_ → ^5^H_3_, ^5^H_6_ (300–330 nm), ^7^F_0,1_ → ^5^L_6_, ^5^D_3_ (360–420 nm), ^7^F_0,1_ → ^5^D_2_ (460–480 nm) and ^7^F_0,1_ → ^5^D_1_ (520–540 nm) transitions. The emission spectrum (*λ*_exc_ = 260 nm) shows several sharp emission peaks between 580 and 630 nm, see Figure 1(F). The emission lines, corresponding to intra configurational 4f^6^ transitions, are assigned to ^5^D_0_ → ^7^F_0_ (580 nm), ^5^D_0_ → ^7^F_1_ (585–600 nm) and ^5^D_0_ → ^7^F_2_ (605–630 nm) transitions. The spectral positions of the luminescence lines of Y_2_O_3_:Eu^3+^ NPs agree well with values reported previously in literature (Li *et al*., 2008).

### Robustness gold‐core rhodamine B‐labelled silica NPs

**Figure 2:**
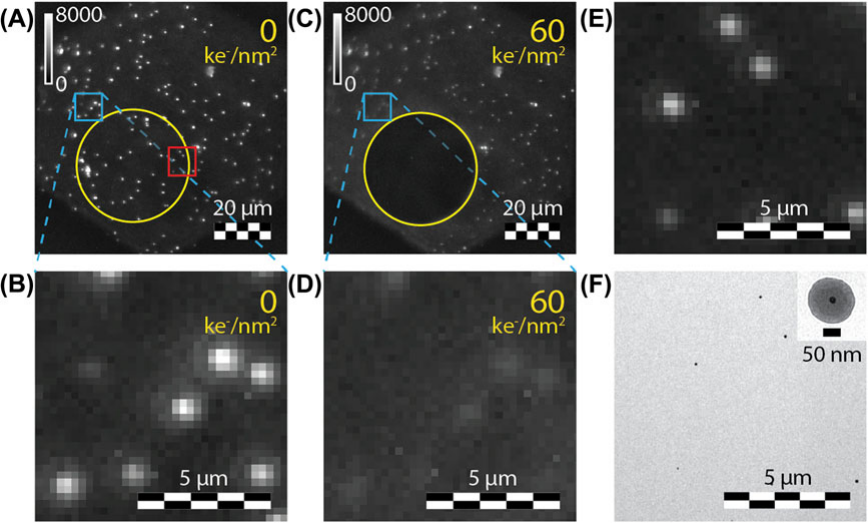
(A)–(E) Luminescence microscope images (*λ*_exc_ = 532 nm) of gold cores coated with rhodamine B‐labelled silica. (A), (B), (E) before and (C), (D) after exposure to an electron dose of 60 ke^−^ nm^–2^. The area inside the yellow circles in (A), (C) was exposed to the electron beam. (B), (D) Zoom in of area indicated by the blue boxes in (A), (C), respectively. (E) Zoom of the red box indicated in (A). (F) TEM image of the particles shown in (E), the inset shows a single gold‐core coated with rhodamine B‐labelled silica.

In order to get insight in the robustness of the various types of NPs, the luminescence intensity of the NPs was measured before and after electron beam exposure. The gold‐core rhodamine B‐labelled silica NPs were excited with a 532 nm laser and the luminescence of rhodamine B was collected from 555 to 615 nm. The luminescence image of the NPs is shown in Figure 2(A). A distribution of white bright spots is observed, originating from the rhodamine B luminescence of molecules inside the silica shell. Next, the sample was exposed to an electron dose of 60 ke^−^ nm^–2^ and the luminescence image was recorded afterwards. The luminescence image of the gold‐core rhodamine B‐labelled silica NPs after electron beam exposure is shown in Figure 2(C). The area exposed to the electron beam is indicated by the yellow circles in Figures 2(A) and (C). The bright white spots observed in the yellow circle before electron exposure are not observed after electron exposure, indicating that the luminescence of NPs irradiated with electrons is completely vanished. The results indicate that the electron beam easily penetrates through the silica shell. As a result, the conjugated π‐system of the organic dye molecules, which is involved in the luminescent transitions, is modified by the electron beam (Egerton *et al*., 2004). Consequently, the luminescence disappears. In addition, the intensity of the bright white spots just outside the yellow circle in Figure 2(A) drops after electron exposure. Figures 2(B) and (D) show zoom ins of these areas that are indicated by the blue boxes in Figures 2(A) and (C), respectively. The drop in luminescence intensity suggests that a tail of the electron beam and/or secondary electrons expose this part of the specimen. The decrease in intensity seems less for large luminescent spots close to the yellow circle, as can be seen by the white spot right to the yellow circle in Figure 2(C). The large size of the bright spot and the presence of luminescence after electron exposure suggest the presence of a cluster of NPs that does not completely lose its luminescence after electron beam exposure.

The NPs observed with light microscopy were correlated with NPs observed with electron microscopy. A zoom in of the area indicated by the red box in Figure 2(A) is shown in Figure 2(E) and shows several bright white spots. The Transmission Electron Microscope (TEM) image of the same area is shown in Figure 2(F) and shows single gold‐core rhodamine B‐labelled silica NPs which reveals that the bright white spots in Figure 2(E) originate from luminescence of single NPs. The two images are clearly correlated, similar patterns of NPs are observed in both images. The inset in Figure 2(F) shows a zoom in on a single NP. The gold‐core, shown by the dark spot in the centre of the NP, can clearly be distinguished from the dye‐labelled silica shell, shown by the lighter outer layer.

### Robustness quantum dots

**Figure 3:**
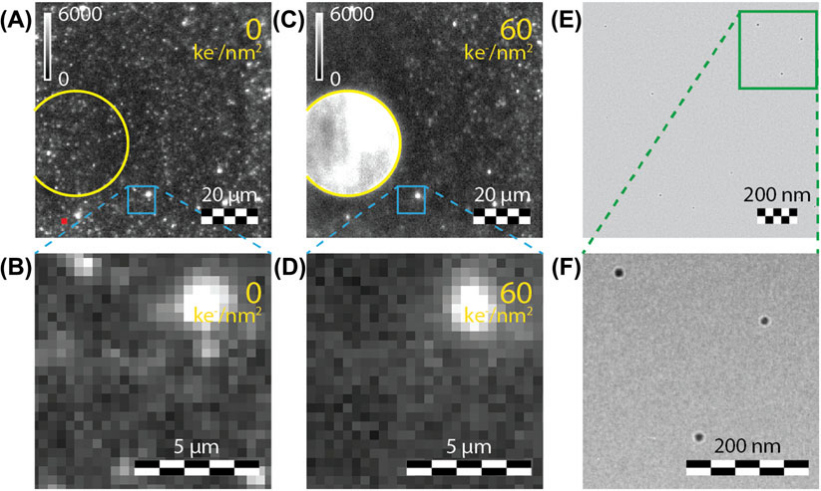
(A)–(D) Luminescence microscope images (*λ*_exc_ = 405 nm) of CdSe/CdS/ZnS core–shell–shell QDs (A), (B) before and (C), (D) after exposure to an electron dose of 60 ke^−^ nm^–2^. The area inside the yellow circles in (A), (C) was exposed to the electron beam. (B), (D) Zoom in of area indicated by the blue boxes in (A) and (C), respectively. (E) TEM image of the area corresponding to the red box in the lower left corner indicated in (A). (F) Zoom of the green box indicated in (E).

The robustness of the second type of NPs, 15 ± 2 nm CdSe/CdS/ZnS core–shell–shell QDs, was investigated next. The QDs were excited at 405 nm and the CdSe exciton emission was collected from 610 to 675 nm. A typical luminescence image is shown in Figure 3(A). Bright luminescent spots are observed over the entire luminescence image, indicating that the QDs are homogeneously spread. In addition, several large bright spots are observed, which probably originate from areas with high local QD concentrations. Next, the QDs were exposed to an electron dose of 60 ke^−^ nm^–2^ and the luminescence of the QDs was measured again. Figure 3(C) shows the luminescence image of the QDs after electron exposure. The area exposed to the electron beam before and after electron bombardment is indicated by the yellow circles in Figures 3(A) and (C). The area inside the yellow circle shows intense luminescence. This luminescence is ascribed to autofluorescence of the organic support. The formation of organic molecules with conjugated double bonds can be induced under electron beam exposure and these molecules are probably formed after the Formvar polymer coated on the TEM grid is exposed to the electron beam. These organic compounds typically absorb in the near ultraviolet (Suzuki, 1967) and emit at longer wavelengths. Moreover, the luminescence from the QDs observed prior to electron exposure is low compared to the autofluorescence, resulting in dominant emission of the polymer species on the grid after electron irradiation. For this reason, the luminescence of the QDs cannot be distinguished from the autofluorescence and the presence of autofluorescence under near UV excitation complicates the analysis of the effect of electron beam exposure on the QD emission. However, QDs that were not directly exposed to the electron beam provide information about the robustness of QDs. Figures 3(B) and (D) show zoom ins of areas adjacent to the area exposed to the electron beam that are indicated by the blue boxes in Figures 3(A) and (C), respectively. The luminescence of most QDs located close to the electron beam is quenched after electron irradiation, just as for the gold‐core rhodamine B‐labelled silica NPs. A bright white spot is still observed after electron exposure, which is probably a large cluster of QDs. The results suggest that the luminescence of QDs directly exposed to the electron beam is also quenched. A possible explanation for the quenching is the creation of structural defects in the QDs by the electron beam. The exciton can be trapped at these defects and subsequently decay nonradiatively. The presence of one or a few defects is enough to quench the luminescence of a single QD (Nirmal & Brus, 1999; De Mello Donega, 2011).

The autofluorescence is more pronounced in the QD sample than in the gold‐core rhodamine B‐labelled silica NP sample. This is related to the difference in measurement conditions. The gold‐core rhodamine B‐labelled silica NPs were excited at 532 nm and the emission was collected from 555 to 615 nm. The QDs were excited at 405 nm and the emission was collected from 610 to 675 nm. Autofluorescence is stronger at UV excitation wavelengths as the absorption of organic molecules on the support is stronger in the UV than in the visible (Suzuki, 1967). After absorption of UV light, emission is observed at lower energies. Part of this emission is positioned in the red and detected in the QD measurements. Absorption at 532 nm is less efficient for the organic compounds and does not lead to strong emission. As a result, the intensity of the autofluorescence is lower in measurements on the gold‐core rhodamine B‐labelled silica NPs.

It was not possible to correlate the QD images obtained with light microscopy with the images recorded with electron microscopy. Figure 3(E) shows a TEM image of the red box in the lower left corner indicated in Figure 3(A). Several single QDs can be observed, although the NPs are not easy to find. For this reason, a zoom in of the area indicated by the green box is shown in Figure 3(F). The luminescence signal of a single QD can be low and in combination with a high background signal, it is difficult to observe single particles. For these reasons, a high concentration of QDs is needed for measurements in our luminescence microscope. Consequently, many QDs are positioned within the diffraction limit and cannot be distinguished in the luminescence image shown in Figure 3(A). It would be interesting to measure clusters of QDs, e.g. supraparticles, since these particles have a higher luminescence signal. This simplifies the correlation between the light and electron microscopy images.

### Robustness lanthanide doped NPs

**Figure 4:**
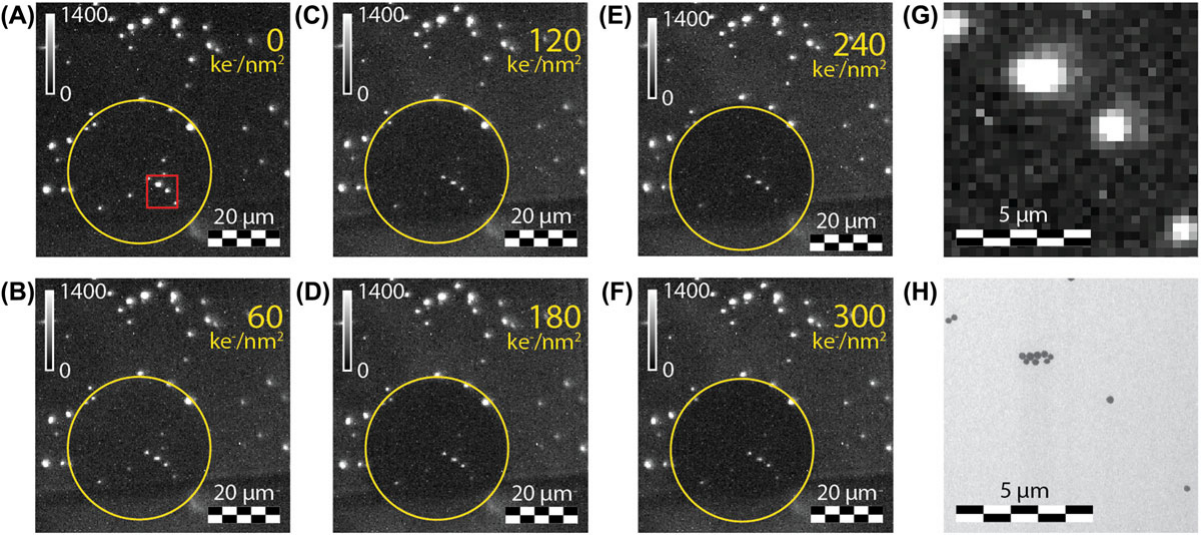
(A)–(G) Luminescence images (*λ*_exc_ = 532 nm) of Y_2_O_3_:Eu^3+^ NPs, (A) and (G) before and (B)–(F) after exposure to an electron dose of (B) 60, (C) 120, (D) 180, (E) 240 and (F) 300 ke^−^ nm^–2^. (G) Zoom of the red box indicated in (A). (H) TEM image of the particles shown in (G).

**Figure 5:**
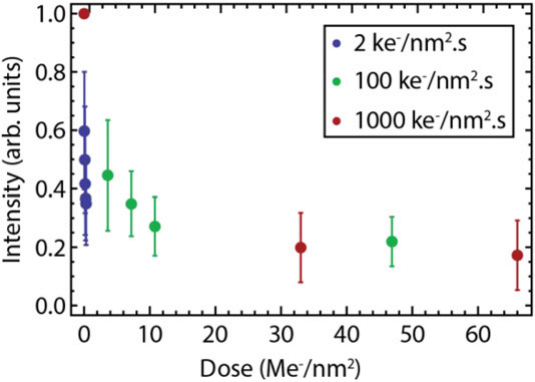
Luminescence intensity of Y_2_O_3_:Eu^3+^ NPs as function of electron dose measured with electron fluxes of 2 ke^−^ nm^–2^ s (blue dots, total dose: 0.3 Me^−^ nm^–2^), 100 ke^−^ nm^–2^ s (green dots, total dose: 47 Me^−^ nm^–2^) and 1000 ke^−^ nm^–2^ s (red dots, total dose: 66 Me^−^ nm^–2^). The electron energy is 120 keV in all experiments. Note that the intensity is normalised on each particle before electron exposure.

To investigate the robustness of 228 ± 23 nm Y_2_O_3_:Eu^3+^ (5%) NPs, the NPs were excited at 532 nm and the emission was collected from 607.5 to 612.5 nm using a narrow band filter. A typical luminescence image of the NPs is shown in Figure 4(A). Several bright spots are observed, originating from ^5^D_0_ → ^7^F_2_ emission of Eu^3+^ ions incorporated in the Y_2_O_3_ NPs. Next, the sample was exposed to an electron dose of 60 ke^−^ nm^–2^ and the luminescence image of the NPs was recorded again. Figure 4(B) shows the luminescence image after electron exposure. The area exposed to the electron beam is again indicated by the yellow circles in Figures 4(A) and (B). In contrast to the gold‐core rhodamine B‐labelled silica NPs and QDs, the bright white spots observed in the yellow circle before electron exposure are still observed after electron bombardment. The results show that the luminescence of Y_2_O_3_:Eu^3+^ NPs survives direct electron beam exposure. To further investigate the robustness of the Y_2_O_3_:Eu^3+^ NPs, the NPs were exposed to four additional electron doses of 60 ke^−^ nm^–2^. In between the electron exposures luminescence images were recorded. Figures 4(A)–(F) show the luminescence images before (a) and after (b‐f) electron exposure with the total electron dose indicated in the upper right corner. The luminescence of the Y_2_O_3_:Eu^3+^ NPs is still observed after an electron dose of 300 ke^−^ nm^–2^. The intensity of the NPs after the various electron doses was calculated using the ThunderSTORM plugin in ImageJ software. This software is typically used to determine the position of single luminescent molecules, but it also calculates the intensities and background levels. The results are shown by the blue dots in Figure 5. A decrease of the luminescence intensity to ∼35% of the initial intensity is observed. The NPs in the area adjacent to the electron beam show bright luminescence after electron beam exposure, in contrast to the gold‐core rhodamine B‐labelled silica NPs and the QDs. The intensity drop after five exposure cycles is only 20%. The Y_2_O_3_:Eu^3+^ NPs show good robustness compared to the gold‐core rhodamine B‐labelled silica NPs and QDs. This can be in part related to the size of the Y_2_O_3_:Eu^3+^ NPs, 228 ± 23 nm, which is significant larger than the 133 ± 6 nm gold‐core rhodamine B‐labelled silica NPs and the 15 ± 2 nm CdSe/CdS/ZnS quantum dots. As a result, the electron beam has to travel a longer distance through the Y_2_O_3_:Eu^3+^ NP to fully penetrate the NP. Monte Carlo simulations (Casino v3.3) were performed to estimate the penetration depths of 120 keV electrons. Simulations obtained considering a 230 nm Y_2_O_3_ sphere with density 5.01 g cm^–3^ and threshold displacement energy of 50 eV (Zinkle & Kinoshita, 1997) show that an electron exits a NP after a few scattering events. This result is consistent with electron penetration depths of 0.1–1.9 μm for acceleration energies of 5–15 keV reported for Y_2_O_3_ bulk materials (Shea & Walko, 1999; den Engelsen *et al*., 2013). In addition, the interaction volume and penetration depth of the electron beam is dependent on the material properties. For example, the interaction volume increases for materials with a lower density (Ortiz *et al*., 1981). Silica and CdS, the main components of the gold‐core rhodamine B‐labelled silica NPs and the CdSe/CdS/ZnS core–shell–shell QDs, have a lower (silica) or similar (CdS) density as Y_2_O_3_. For this reason, it is concluded that the electron beam penetrates completely through all types of NPs investigated here. The observation of clear NP luminescence from Y_2_O_3_:Eu^3+^ after multiple exposures to 60 ke^−^ nm^–2^ indicates that the Y_2_O_3_:Eu^3+^ NPs are more stable than the gold‐core rhodamine B‐labelled silica NPs and quantum dots.

The Y_2_O_3_:Eu^3+^ images obtained with light microscopy were correlated with the images measured with electron microscopy. Figure 4(G) shows a zoom in of the area indicated by the red box in Figure 4(A). Several bright luminescent spots of various size and intensity are observed. The corresponding TEM image is shown in Figure 4(H) and shows single and groups of NPs. Similar patterns of NPs are observed in both images, showing a clear correlation between the images.

The results indicate that Y_2_O_3_:Eu^3+^ NPs remain luminescent after exposure to high electron doses. To study the robustness of these NPs further, measurements were performed with higher electron fluxes. Again, the sample was exposed to the electron beam and the luminescence intensity was measured before and in between exposures. Figure 5 shows the intensity of the NPs as function of electron dose measured with electron fluxes of 2, 100 and 1000 ke^−^ nm^–2^ s. For the two highest fluxes, an initial drop in intensity is observed followed by a rapid stabilisation of the intensity. Note that in our measurements the total dose obtained with the lowest flux, i.e. 2 ke^−^ nm^–2^ s, is significantly lower than the dose obtained with the two higher fluxes. However, the intensity loss measured with the lowest electron flux is similar to the intensity reduction measured with an electron flux of 100 ke^−^ nm^–2^ s after the same overall electron dose. In addition, the luminescence intensity after a specific dose is approximately similar for electron fluxes of 100 and 1000 ke^−^ nm^–2^ s. These results indicate that the intensity loss does not depend on the electron flux, but on the total electron dose. The NPs preserve ∼20% of the initial luminescence intensity, up to electron doses of 66 Me^−^ nm^–2^.

It is interesting to try and understand the mechanism responsible for the luminescence intensity loss after electron exposure. Y_2_O_3_:Eu^3+^ microcrystals are expected to be stable as they are analogous to Y_2_O_2_S:Eu^3+^ microcrystals that have been used for decades as red phosphor in cathode ray tubes because of their bright luminescence and resistance to prolonged electron exposure (Hase *et al*., 1990; Blasse & Grabmaier, 1994). However, the size of the 230 nm Y_2_O_3_:Eu^3+^ NPs measured in this report is significantly smaller than the ∼5 μm crystals used in cathode ray tubes. Our results could indicate that size and surface to volume ratio are important parameters for the luminescence stability. It is suggested that the core of the NPs is stable and hardly affected by the electron beam, while the surface layers are less stable under electron bombardment. This can be explained by two phenomena. First, atoms at the surface of the host lattice usually have a lower degree of crystallinity (ordering) than atoms inside the NP (Wei *et al*., 2002; Van Hest *et al*., 2017). Consequently, surface atoms are easier distorted from their equilibrium position and more structural defects are created in the surface layers than in the interior of the NP when exposed to the electron beam. Second, the surface of the NP can be covered with reactive species such as adsorbed water or carbon monoxide after sample preparation. The electron beam can activate a reaction with these molecules resulting in the creation of defects in the outer layers of the NPs. In both cases, defects are created in the host material which can quench the luminescence of nearby Eu^3+^ ions. The localised character of transitions of Eu^3+^ ions hamper quenching by defects at larger distances from the defects (Buijs *et al*., 1987). Consequently, the europium ions at the surface of the NP are quenched after electron bombardment, while the luminescence of Eu^3+^ ions in the core of the NPs remains intact. For surface‐related losses, a loss of 80% in luminescence intensity would imply that the luminescence in the ∼50 nm outer shell of the NPs is quenched.

Another possible explanation for the decrease in intensity after electron bombardment is carbon deposition on the NPs. The electron beam reacts with hydrocarbons present in the TEM chamber. As a result, hydrocarbon ions are created which condense on the irradiated area (Kumao *et al*., 1981; Egerton *et al*., 2004). The carbon layer can react with the NPs, which results in a lower luminescence intensity. Based on the experimental results, it is not possible to unravel in detail the mechanism responsible for the luminescence intensity loss of the NPs after electron bombardment. The present study clearly demonstrates that Y_2_O_3_:Eu^3+^ NPs are robust to electron doses up to 66 Me^−^ nm^–2^.

The results presented demonstrate the variation in robustness to electron bombardment of various types of NPs. The luminescence of gold‐core rhodamine B‐labelled silica NPs and CdSe/CdS/ZnS core–shell–shell QDs is completely quenched after one cycle of electron bombardment. In contrast, Y_2_O_3_:Eu^3+^ NPs are robust to electron exposure and show luminescence after five cycles of electron bombardments. The luminescence intensity initially drops, but rapidly reaches a constant value. The intensity loss is not dependent on the electron flux of the electron beam, but on the total electron dose. The reduction in intensity is probably caused by quenching of Eu^3+^ ions in the outer layers of the NP after the creation of defects or by the interaction with carbon which deposits on the NP during electron exposure. Due to the large differences in size between the Y_2_O_3_:Eu^3+^ NPs and QDs differences in quenching behaviour are expected. It would be particularly interesting to investigate the quenching behaviour of larger QD based particles. Examples of such particles include QDs with a thicker shell or supra particles of QDs (Montanarella *et al*., 2017). The luminescence of single supra particles of QDs should be clearly visible.

Smaller NPs, e.g. with a diameter up to 50 nm, are interesting for the study of biological samples, since they can enter cells via active uptake (Shang *et al*., 2014). For this reason, it is interesting to investigate the robustness of smaller Y_2_O_3_:Eu^3+^ NPs. Note that the luminescence intensity decreases linearly with the number of luminescent ions in the NP and thus with the volume of the NP and a tradeoff between luminescence intensity and size should be considered. Interestingly, the narrow and characteristic emission lines of lanthanide ions can be used to create a large number of unique luminescent labels by changing and combining the type of lanthanide dopant. The luminescent labels can be coupled to a specific antibody in order to follow the binding of a variety of molecules to specific sites in the cell by measuring the emission spectrum of the luminescent label, even down to the single particle level. Finally, we note that the Y_2_O_3_:Eu^3+^ NPs are particularly interesting for applications involving cathode luminescence (Furukawa *et al*, 2013; Morrison *et al*., 2015; Nagarajan *et al*., 2016).

## Conclusions

The robustness of various types of nanoparticles (NPs), namely gold‐core rhodamine B‐labelled silica, CdSe/CdS/ZnS core–shell–shell QDs and Y_2_O_3_:Eu^3+^ (5%), was investigated by recording the luminescence of (single) NPs before and after various cycles of electron bombardments. The luminescence of the rhodamine B dye and QDs was completely quenched after a single exposure to 60 ke^−^ nm^–2^ with energy of 120 keV, while the Y_2_O_3_:Eu^3+^ NPs are robust, i.e. luminescence is observed after exposure to 120 keV electrons, even after an electron dose of 66 Me^−^ nm^–2^. The intensity of Y_2_O_3_:Eu^3+^ NPs initially decreases, but rapidly reaches a constant value. Approximately 20% of the original intensity is maintained. The intensity loss is independent on the electron flux and determined by the total electron dose. The cause of the drop in intensity is probably the creation of defects in the surface layers of the NP or the interaction with carbon deposits accumulating on the NPs during electron beam exposure.

The robust character of the Y_2_O_3_:Eu^3+^ makes the NPs promising as fiducial marker in correlative light and electron microscopy. In addition, the sharp and characteristic line emission can be used to create a variety of luminescent labels by changing and combining the type of dopant ion. The various labels can be detected down to the single particle level by recording the emission spectra of these labels.
